# Optimization of Variable Ventilation for Physiology, Immune Response and Surfactant Enhancement in Preterm Lambs

**DOI:** 10.3389/fphys.2017.00425

**Published:** 2017-06-23

**Authors:** Erzsébet Bartolák-Suki, Peter B. Noble, Samer Bou Jawde, Jane J. Pillow, Béla Suki

**Affiliations:** ^1^Department of Biomedical Engineering, Boston UniversityBoston, MA, United States; ^2^Anatomy, Physiology and Human Biology, School of Human Sciences, University of Western AustraliaPerth, WA, Australia; ^3^Centre of Neonatal Research and Education, Pediatrics, Medical School, University of Western AustraliaPerth, WA, Australia

**Keywords:** compliance, surfactant protein, inflammation, alveolar stability

## Abstract

Preterm infants often require mechanical ventilation due to lung immaturity including reduced or abnormal surfactant. Since cyclic stretch with cycle-by-cycle variability is known to augment surfactant release by epithelial cells, we hypothesized that such *in vivo* mechanotransduction improves surfactant maturation and hence lung physiology in preterm subjects. We thus tested whether breath-by-breath variability in tidal volume (V_T_) in variable ventilation (VV) can be tuned for optimal performance in a preterm lamb model. Preterm lambs were ventilated for 3 h with conventional ventilation (CV) or two variants of VV that used a maximum V_T_ of 1.5 (VV1) or 2.25 (VV2) times the mean V_T_. V_T_ was adjusted during ventilation to a permissive pCO_2_ target range. Respiratory mechanics were monitored continuously using the forced oscillation technique, followed by postmortem bronchoalveolar lavage and tissue collection. Both VVs outperformed CV in blood gas parameters (pH, *Sa*O_2_, cerebral O_2_ saturation). However, only VV2 lowered PaCO_2_ and had a higher specific respiratory compliance than CV. VV2 also increased surfactant protein (SP)-B release compared to VV1 and stimulated its production compared to CV. The production and release of proSP-C however, was increased with CV compared to both VVs. There was more SP-A in both VVs than CV in the lung, but VV2 downregulated SP-A in the lavage, whereas SP-D significantly increased in CV in both the lavage and lung. Compared to CV, the cytokines IL-1β, and TNFα decreased with both VVs with less inflammation during VV2. Additionally, VV2 lungs showed the most homogeneous alveolar structure and least inflammatory cell infiltration assessed by histology. CV lungs exhibited over-distension mixed with collapsed and interstitial edematous regions with occasional hemorrhage. Following VV1, some lambs had normal alveolar structure while others were similar to CV. The IgG serum proteins in the lavage, a marker of leakage, were the highest in CV. An overall combined index of performance that included physiological, biochemical and histological markers was the best in VV2 followed by VV1. Thus, VV2 outperformed VV1 by enhancing SP-B metabolism resulting in open alveolar airspaces, less leakage and inflammation and hence better respiratory mechanics.

## Introduction

Respiratory distress syndrome (RDS) or hyaline membrane disease occurs in preterm (<37 weeks' gestation) and in some term babies when type II alveolar epithelial (AEII) cells produce insufficient surfactant (Stevens et al., 2007; Engle, 2008). Surfactant deficiency is usually primary (Verma, 1995). However, secondary surfactant deficiency with prior normal surfactant synthesis also occurs due to hemorrhage, pneumonia, sepsis, hypoxia, and ventilator-induced lung injury or surfactant inhibition by substances reaching the alveoli (Herting et al., 2000; Kneyber et al., 2005).

Surfactant is a complex mixture of lipids and proteins lining the internal surface of the lung. By reducing surface tension at the air-liquid interface, surfactant is critical for lung stability and hence life (Veldhuizen et al., 1998). Because of the high surface tension in RDS, the lungs cannot inflate and patients invariably require mechanical ventilation often supplemented with exogenous surfactant and/or high oxygen levels (Stevens et al., 2007). Despite some successes in exogenous surfactant therapy, many problems remain. For example, the spatial distribution of exogenous surfactant introduced into the lungs is unknown and difficult to regulate. It is likely that the collapsed lung regions in RDS receive little surfactant, remain collapsed, and fail to improve gas exchange. Differences between natural and synthetic surfactants applied in these therapies pose additional challenges (Engle, 2008). Natural surfactant contains surfactant proteins that play crucial roles in normal surfactant functioning (Ingenito et al., 1999). For example, surfactant protein (SP)-B is essential to reducing surface tension at the air-liquid interface and enabling breathing. SP-C has a similar role in surface layer mechanics while SP-A and SP-D support the innate defense of the lung (Johansson and Curstedt, 1997). However, extracting good quality surfactant in sufficient quantity is difficult and expensive. Synthetic surfactants are a simplified mixture of lipids and most lack surfactant proteins. Some recombinant proteins reminiscent of natural surfactant proteins are available, but their functionality does not reach that of the natural proteins (Been and Zimmermann, 2007). It is noteworthy, however, that newer synthetic surfactants such as CHF 5633 that contain SP-B and SP-C analogs, significantly improved oxygenation and ventilation efficiency in a premature lamb model of surfactant deficiency (Seehase et al., 2012), whereas KL4 that includes only an SP-B analog, attenuated lung inflammation (Wolfson et al., 2012).

Nevertheless, there is also a need for clinical treatments that induce endogenous surfactant production and secretion in the lung under conditions requiring surfactant supplementation in newborn infants. Potential candidate drugs are proposed (Blanco et al., 2004), but the most important known endogenous surfactant stimulator is mechanical stretching of the lung (Wirtz and Dobbs, 1990). Our previous data suggest that monotonous cyclic stretching of surfactant secreting cells in culture down-regulates surfactant secretion (Arold et al., 2009). However, normal surfactant secretion is restored when cells are stretched using a variable stretch pattern, in which the amplitude of stretch is varied randomly on a cycle-by-cycle basis, (Arold et al., 2009). We and others also found that a variable ventilation (VV) pattern, in which tidal volume (*V*_T_) is varied on a cycle-by-cycle basis, improves gas exchange, reduces ventilator-induced lung injury (Lefevre et al., 1996; Mutch et al., 2000a,b; Arold et al., 2002, 2003; Bellardine et al., 2006; Thammanomai et al., 2008; Spieth et al., 2009) and increases the alveolar surfactant pool (Arold et al., 2003) in adult animals. These findings raise the possibility that variability in stretch directly delivered to cells *in vivo* could also stimulate endogenous surfactant production and secretion in preterm infants suffering from RDS.

Since VV in preterm lambs improves lung physiology compared to CV without introducing lung inflammation (Pillow et al., 2011; Berry et al., 2012), we hypothesized that improvements in lung physiology during VV in surfactant deficient lungs are due to enhanced surfactant production and secretion. To test this hypothesis, we investigated whether features of VV can be optimized to enhance surfactant metabolism and lung function in a preterm lamb model neonatal RDS.

## Methods

### Animal care and preparation

Animal studies were performed at The Large Animal Facility, at the University of Western Australia as described previously (Berry et al., 2012). The protocol was approved by the Institutional Animal Ethics Committee of both Boston University and the University of Western Australia. Merino ewes were exposed to betamethasone 2 days prior to delivery. Ewes received medroxyprogesterone (150 mg, IMI, Pfizer, Australia) at 114 d gestation prior to betamethasone (5.7 mg, IMI, Shering Plow, Sydney, Australia) at 126 and 127 day gestation. Ewes were premedicated at 128 day gestation (term = 150 day) with acepromazine 0.05 mg/kg (Ceva, Australia); buprenorphine 0.01 mg/kg (Reckitt and Coleman, Australia) before induction with ketamine (5 mg/kg, IV, Pfizer, Australia) and midazolam (0.25 mg/kg, IV, Alphapharm, Australia) and intubated. Anesthesia was maintained subsequently with isofluorane (2–3%) in oxygen.

Fetuses were exteriorized via a hysterotomy and catheters were inserted into the right external carotid and right jugular vein for monitoring and arterial blood gas sampling. Lambs were intubated with a cuffed tracheal tube (4.5 mm i.d.) after exteriorization of the head and neck and lung fluid was suctioned. The lambs were delivered, weighed and placed on pre-assigned ventilation protocols (see below). Propofol (0.1 mg/kg/min, Repose™, Norbrook Laboratories, Victoria Australia) and remifentanil (0.05 μg/kg/min, Ultiva™, Glaxo Smith Kline, Victoria, Australia) were administered via continuous infusion through a jugular venous catheter for anesthesia, analgesia and suppression of spontaneous breathing. Arterial blood gases including partial pressure of oxygen (*Pa*O_2_) and carbon dioxide (*P*aCO_2_), and arterial oxygen saturation (*Sa*O_2_) were obtained at 15 min intervals for the first 60 min followed by hourly collection from an umbilical (postductal) arterial catheter blood sample (Rapidlab 1265, Siemens Healthcare, Vic, Australia). Cerebral tissue oxygen saturation (CerStO_2_) was obtained using Near Infrared Spectroscopy monitoring (CASMED, Inc, Branford, Conn, USA). Pulse rate and postductal oxyhemoglobin saturation (SpO_2_) were monitored continuously using pulse oximetry (N-65, Covidien, Boulder, CO, USA).

### Ventilation protocol

**Figure 1:**
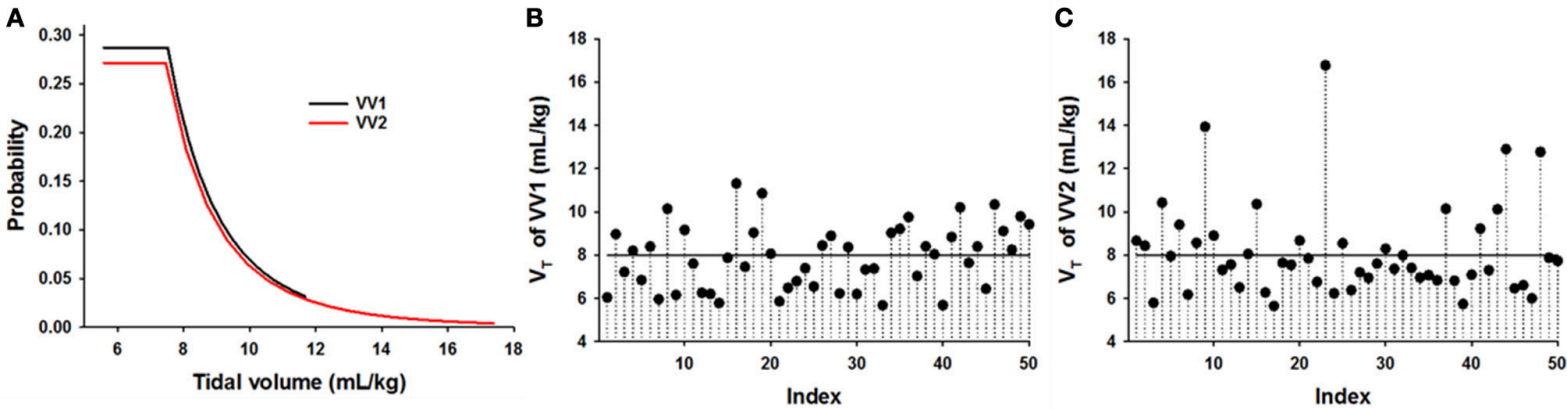
Comparison of two ventilation patterns applied in preterm lambs. **(A)** Probability distribution functions of variable ventilations (VV) in which the maximum tidal volume was 1.5 times the mean tidal volume (V_T_) (VV1, black) or 2.25 times the mean V_T_ (VV2, red). Notice that the tail of the distribution in VV2 is longer than that of VV1 which means that VV2 occasionally includes much larger V_T_ than VV1 even though the mean in both cases was 8 mL/kg. **(B,C)** Realizations of the time series of VV1 and VV2, respectively, showing 50 consecutive V_T_'s. The solid line represents conventional ventilation (CV).

Lambs were ventilated at a PEEP of 5 cmH_2_O and a frequency of 50 breaths per minute for CV and a mean frequency of 50 breaths per min for VV with rates varying between 30 and 70 breath/min for 3 h with CV (*n* = 5) or one of two variants of VV using the Flexivent ventilator/oscillator system (SciReq, Montreal Ca). The distributions of *V*_T_ were designed as previously described (Thammanomai et al., 2008). Briefly, the distribution has a flat part for V_T_ values lower than the mean *V*_T_ followed by a hyperbolically decreasing part up to a maximum V_T_. In VV1 (*n* = 6), the maximum *V*_T_ was 1.5 times the mean *V*_T_ whereas in VV2 (*n* = 7), it was 2.25 times the mean *V*_T_ allowing larger variations in *V*_T_ (Thammanomai et al., 2008). Figure 1A compares the distributions and Figures 1B,C demonstrate realizations of VV1 and VV2, respectively. Ventilation started with a *V*_T_ of 8 mL/kg in the CV group and a mean *V*_T_ of 8 mL/kg in the VV groups. During the 3 h ventilation, target *V*_T_ or mean *V*_T_ was adjusted to approximately maintain *P*aCO_2_ between 40 and 50 mmHg. Blood gases were assessed initially at 15 min intervals for 1 h followed by measurements at 30 min intervals. If *P*aCO_2_ increased beyond 50 mmHg, *V*_T_ was increased by 0.5 or 1 mL/kg for CV and in the case of VV, the mean *V*_T_ was increased similarly.

### Respiratory mechanical measurements

Cylinder displacement volume, cylinder pressure and airway opening pressure were recorded by the Flexivent and used to calculate tracheal pressure and *V*_T_ after correction for the impedance of the tubing derived from calibration immediately prior to ventilation of each lamb. Respiratory mechanics were assessed using the low-frequency forced oscillation technique (Bates et al., 2011), obtained at intervals to coincide with blood gas measurements. Tracheal pressure and flow signals were collected during an optimized ventilator waveform containing frequencies between 0.5 and 13 Hz delivered by the FlexiVent (Lutchen et al., 1993). The peak-to-peak volume of the oscillatory signal was matched to the *V*_T_ used to ventilate the lamb just prior to the forced oscillation measurement. Respiratory input impedance was calculated as the ratio of the cross-power spectrum of pressure and flow and the auto-power spectrum of flow. The constant phase model (Hantos et al., 1992) was fitted to the impedance spectra to determine mechanical parameters including Newtonian resistance, tissue damping and tissue elastance (*H*). The mechanical condition of the lung was represented by the respiratory compliance (*C* = 1/*H*) and the specific compliance *C*_s_ = *C*/W where W is the body weight at birth. The alveolar-arterial (A-a) gradient was computed from the blood gas values as ${\text{FiO}}_{2}^{*}$(*P*_atm_-*P*_H2O_)-*Pa*CO_2_/0.8-*Pa*O_2_, where FiO2 is the inspired fraction of O_2_, *P*_atm_ is ambient atmospheric pressure and *P*_H2O_ is the partial pressure of water vapor taken as 47 mmHg.

### Biochemistry

At study completion, lavage and lung tissue samples were collected for biochemistry (n_CV_ = 5, n_VV1_ = 6 and n_VV2_ = 7) and histology. BCA protein assay (Pierce, Rockford, IL) was used to determine protein concentrations. Equal amounts of protein (4.2 μg for lavage and 3.7 μg for lung) were processed with reducing sodium dodecyl sulfate polyacrylamide gel electrophoresis (SDS-PAGE) using precast 4–20% gradient gels (BioRad Laboratories, Hercules, CA). Following electrophoresis, proteins were transferred to polyvinylidene fluoride membranes (Millipore, Bedford, MA) and blocked overnight in 5% milk (BioRad Laboratories, Hercules, CA) in phosphate buffered saline containing 0.05% Tween 20. Western blot analyses were performed with sheep specific anti-surfactant proteins (SP)-A (AB3424, CHEMICON Int. Inc. Billerica, MA), SP-B (ab40876, Abcam Inc. Cambridge, MA), SP-C (LS-B4588, LifeSpan Biosciences, Inc. Seattle, WA) and SP-D (ab17781 Abcam Inc. Cambridge, MA) antibodies, for cytokines with anti-sheep tumor necrosis factor (TNF)α (MCA906GA), anti-sheep interleukin-1 (IL-1)β (AHP423) (both antibodies from BioRad Laboratories, Hercules, CA), and for plasma proteins with anti-IgG heavy and light chains (ab85791 and ab133470, Abcam Inc. Cambridge, MA). Monoclonal anti-β actin antibody (ab8226, Abcam Inc. Cambridge, MA) was used as a loading control. Primary and secondary antibody incubation was done for 1 h. Quantitative densitometry was performed after chemiluminescence detection using Super Signal West Pico chemiluminescence substrates (Pierce, Thermo Fisher Scientific Inc. Waltham, MA).

### Histology

Left upper lobes of the lungs (*n* = 5 in each group) were fixed in 10% formalin (neutral buffered) at a pressure of 30 cmH_2_O for ~96 h and paraffin-embedded. Coronal sections (7 μm) were obtained and deparaffinized in xylene and rehydrated in decreasing alcohol dilution series. Routine H&E staining was performed using the Protocol Reagents (Fisher Scientific, Waltham, MA) to visualize alveolar morphology. Phospholipids were visualized by Pearse's staining method and lipids by Nile Blue A staining using kits from Electron Microscopy Sciences (Hartfield, PA). Histological analysis was carried out blindly under light microscopy (Nikon Eclipse 50i microscope and SPOT camera, Micro Video Instruments, Avon, MA) at low (10X), medium (40X), and high (100x) magnifications for whole lung sections and 20 sections/lamb were chosen randomly for analysis. The degree of ventilation induced injury was scored in whole sections using criteria derived from previous histological investigations (Muscedere et al., 1994; Hamakawa et al., 2011). Specifically, scores between 0 to 10 were assigned to each of the following characteristics: (1) *Alveolar wall thickening* reflects structural changes in the alveolar extracellular matrix (ECM) due to swollen and/or loose ECM and hyaline membrane formation covering the epithelial layer; (2) *Alveolar wall rupture* representing thinner or broken alveolar walls with tissue retraction; (3) *Blood cell infiltration* demonstrates an increase in inflammatory cell numbers in the alveolar wall; (4) *Interstitial edema* reflects proteins containing free fluid and swelling of the alveolar wall space; (5) *Vascular bed injury* presents degeneration of vessels (swollen, vacuole rich vascular walls with cell necrosis); and (6) *Alveolar wall flooding* represents platelet invasion including infarcts such as hemorrhagic changes of alveolar walls after vascular rupture. Lipid containing cells were scored based on both Pearse's and Nile Blue A staining to evaluate the overall size and distribution of lipid staining. Scores were between 0 and 10 with 0 representing no phospholipid positive cells per microscopic field, whereas 10 representing 10 positive cells per field at a magnification of 100x.

### Performance indexes

Three different performance indices were calculated from the data: (1) a physiology index (PI) based on *Pa*CO_2_, *Pa*O_2_, SaO_2_, *V*_T_, FiO_2_, CertSO_2_, and *C*_*s*_; (2) a histology index (HI) based on the lipid staining scores and the histology injury scores that included alveolar wall thickening, alveolar wall breakage, blood cell infiltration, interstitial edema, vascular bed injury, and alveolar wall flooding; and (3) a biochemistry index (BI) based on SP-A, SP-B and SP-D in both lung tissue and the bronchoalveolar lavage, IL-1β and TNFα in tissue and lavage, as well as IgG heavy and light chains in the lavage. Since the physiological parameters were measured at many time points, PI was evaluated at each time point, whereas HI and BI were only obtained at 180 min. The performance index was computed as the average of individual parameters each normalized by the mean of the corresponding parameter of the CV group. For example, in a given lamb at a single time point, *Pa*O_2_, *Pa*CO_2_, etc. were normalized with the mean of *Pa*O_2_, *Pa*CO_2_ of the CV group at 45 min. These normalized parameters were then averaged to define the PI. If a higher value of a parameter indicates a deterioration in function (e.g., lower values of *Pa*CO2 represent better function), then the reciprocal of the parameter was included in PI. Thus, an increase in PI in time and/or with a ventilation above 1 represents improved overall physiology. SP-D is known to be upregulated in the presence of inflammation (Gaunsbaek et al., 2013); hence, the reciprocal of its level was included in the calculation. Since SP-C is involved both in the regulation of surface tension and immunomodulatory responses of the lung (Mulugeta and Beers, 2006), proSP-C was not included in the biochemistry index. Finally, the PI (taken at 180 min), the histology index and the biochemistry index were used to define a Combined Index (CI) to characterize the performance of 180 min of ventilation. In BI and HI mean values were used. Thus, PI, the histology and biochemistry indices contribute with equal weight to the final value of CI. Table 1 summarizes how the indexes were calculated.

**Table 1:** Definition of physiology, histology, biochemistry, and combined indexes.

**Index**	**Number of parameters**	**Parameters (P)**	**time point (s) (min)**	**Equation**
Physiology Index (PI)	7	*Pa*CO_2_, *Pa*O_2_, SaO_2_, VT, FiO_2_, CertSO_2_, and C_s_	45-180	$PI=\frac{1}{7}\times \overset{7}{\underset{1}{\sum }}{\left(physiology\;parameter\right)}^{\alpha }$
Histology Index (HI)	7	Alveolar wall thickening, alveolar wall breakage, blood cell infiltration, interstitial edema, vascular bed injury, alveolar wall flooding, and phospholipid in cells	180	$HI=\frac{1}{7}\times \overset{7}{\underset{1}{\sum }}\left(10-\bar{histology\;parameter\;score}\right)$
Biochemistry Index (BI)	13	SP-A, SP-D, IL-1β and TNFα in tissue and lavage, SP-B in tissue, SP-B pro and SP-B dimer in lung, IgG heavy and light chains in tissue	180	$BI=\frac{1}{13}\times \overset{13}{\underset{1}{\sum }}{\left(\bar{biochemistry\;parameter}\right)}^{\alpha }$
Combined Index (CI)	3	PI at 180 min, HI, and BI	180	$CI=\frac{PI\left(time=180\right)+HI+BI}{3}$

*α = 1 if higher paramter value signifies an improvement*.

*α = −1 if higher parameter value signifies a deterioration*.

### Data analysis and statistics

The data were analyzed by two-way repeated measure ANOVA to assess any time dependence as well as ventilation mode dependence on the physiology parameters. The Holm-Sidak *post-hoc* pairwise comparison was applied with adjusted *p*-values to control for the increased likelihood of type I error. The 180 min data were also analyzed by one way ANOVA followed by Holm-Sidak or Dunn's *post-hoc* pairwise comparisons for normally and not normally distributed data sets, respectively. Data that were not normally distributed were analyzed either after log transformation (two-way ANOVA) or using ANOVA on ranks. All statistical analyses were performed in SigmaPlot (Systat Software, Inc.). Significance was accepted at the level of 0.05, except for the *post-hoc* analysis following two-way ANOVA.

## Results

### Variable ventilation outperforms conventional ventilation in physiology parameters

**Figure 2:**
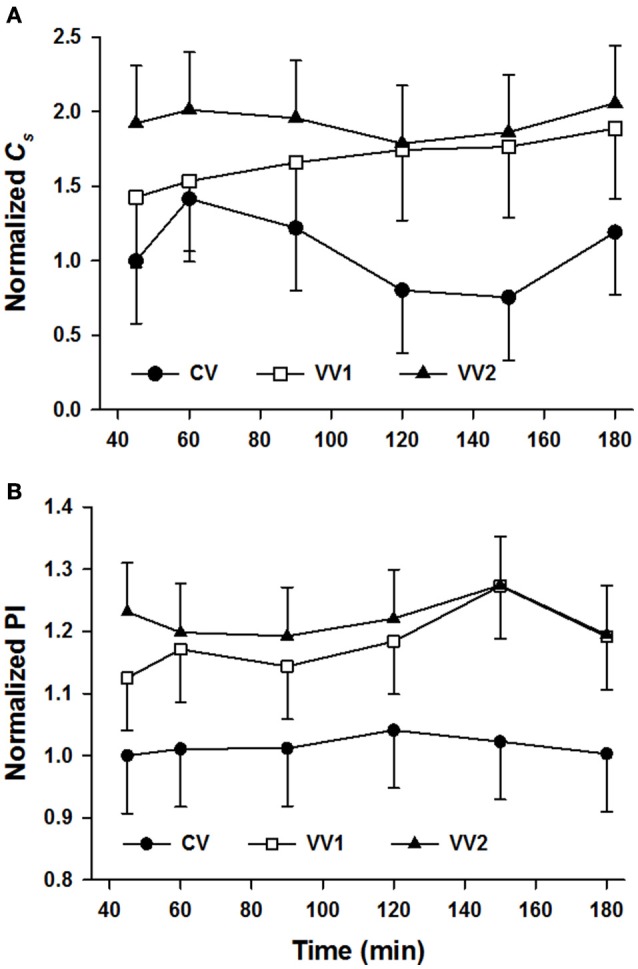
Mean and standard deviation (SD) of the specific compliance (*C*_s_) and the physiology index (PI) both normalized as a function of time in preterm lambs ventilated with conventional ventilation (CV), variable ventilation with maximum tidal volume of 1.5 times the mean (VV1) and another version of variable ventilation in which maximum tidal volume was 2.25 times the mean tidal volume (VV2) as defined in Figure 1. Data are normalized to unity with the mean of the CV group at the first time point. **(A)** The difference in the means of the specific compliance among the different levels of ventilation is statistically significant (*p* = 0.027 independent of time) with values larger during VV2 than CV. **(B)** The difference in the means of PI among the different levels of ventilation is statistically significant (*p* < 0.001 independent of time) with values larger during both VV1 and VV2 than CV.

All physiological variables were assessed at 15 min and/or 30 min into the ventilation protocol. Most variables showed no statistically significant differences between groups due to large intra- and inter-group variabilities. Thus, only data between 45 and 180 min were analyzed further. Table 2 summarizes the physiology parameters averaged over time. Both VV1 and VV2 had better results than CV for pH, *Pa*O2, SaO2 and CerStO2, but VV1 and VV2 did not differ from each other. VV1 also required a lower *V*_*T*_ than CV. VV1 and VV2 did not differ for any of the variables including *Pa*CO_2_, FiO_2_, *C, C*_*s*_, and A-a gradient. However, the improvement of VV2 over CV was much stronger than that of VV1 over CV in several variables (e.g., *P*aCO_2_ and *C*_s_. Figure 2A shows *C*_s_ as a function of time for all three ventilation groups. Figure 2B shows that independent of time, both VV1 and VV2 outperformed CV in terms of PI that characterizes overall lung physiology (*p* < 0.001).

**Table 2:** Mean and standard error of mean (SEM) values of all physiology variables averaged across time for three ventilation modes used to ventilate preterm lambs.

**Variable**	**Ventilation**	***n***	**Mean (sEM)**	***P*****-value (Holm-Sidak)**		
				**CV vs. VV1**	**CV vs. VV2**	**VV1 vs. VV2**
pH	CV	5	7.186 (0.024)	<**0.001**	<**0.001**	0.345
	VV1	6	7.305 (0.022)			
	VV2	7	7.334 (0.021)			
*P*aCO_2_	CV	5	75.8 (5.1)	0.035	**0.003**	0.398
	VV1	6	61.0 (4.6)			
	VV2	7	55.7 (4.3)			
*P*aO_2_	CV	5	42.1 (3.9)	**0.002**	<**0.001**	0.817
	VV1	6	59.4 (3.6)			
	VV2	7	60.5 (3.3)			
SaO_2_	CV	5	82.9 (1.7)	<**0.001**	<**0.001**	0.875
	VV1	6	93.8 (1.5)			
	VV2	7	94.1 (1.4)			
*V*_T_	CV	5	11.6 (0.3)	<**0.001**	0.062	0.047
	VV1	6	10.3 (0.2)			
	VV2	7	11.0 (0.2)			
FiO_2_	CV	5	67.4 (5.9)	0.025	0.026	0.917
	VV1	6	49.1 (5.4)			
	VV2	7	49.8 (5.0)			
CerStO_2_	CV	5	73.1 (2.3)	**0.002**	**0.004**	0.720
	VV1	6	82.9 (2.1)			
	VV2	7	81.8 (1.9)			
C	CV	5	1.5 (0.3)	0.039	0.021	0.970
	VV1	4	2.4 (0.3)			
	VV2	7	2.4 (0.2)			
C_s_	CV	5	0.47 (0.09)	0.030	**0.011**	0.865
	VV1	4	0.73 (0.08)			
	VV2	7	0.75 (0.07)			
A-a gradient	CV	5	344 (41)	0.020	0.028	0.827
	VV1	6	214 (37)			
	VV2	7	225 (34)			

*The p-values in bold represent statistically significant differences using the Holm-Sidak test. PaCO_2_, arterial partial pressure of CO_2_ (mmHg); PaO_2_, arterial partial pressure of O_2_ (mmHg); SaO_2_, arterial oxygen saturation (%); V_T_, tidal volume (mL); FiO_2_, fraction of inspired O_2_ (%); CerStO_2_: cerebral tissue oxygen saturation (%); C, respiratory compliance (mL/cmH_2_O); C_s_, specific compliance (mL/cmH2O/kg); A-a gradient, alveolar to arterial gradient; CV, conventional ventilation; VV1 and VV2, variable ventilations as defined in Figure 1*.

### Surfactant protein (SP) metabolism is ventilation mode dependent

**Figure 3:**
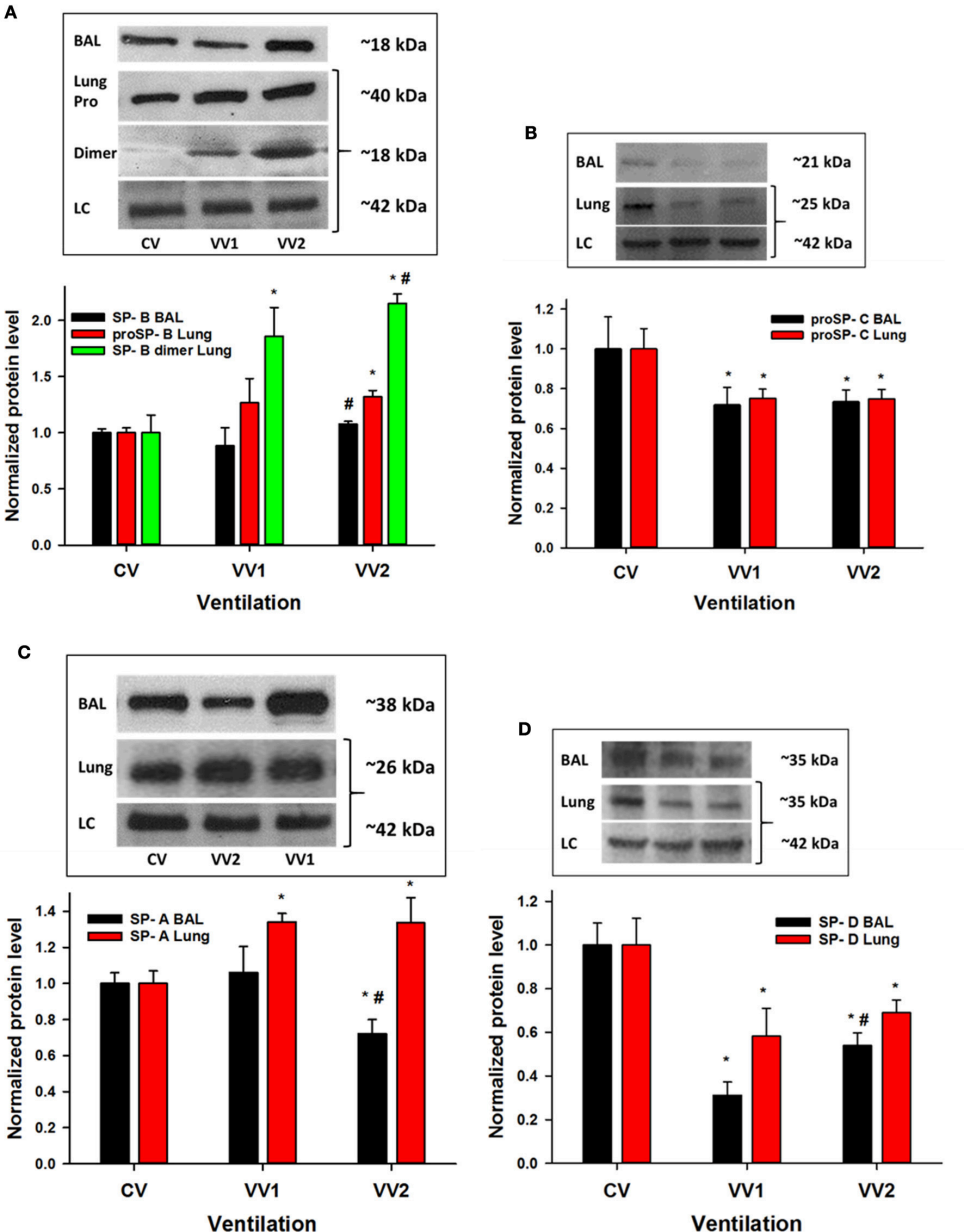
Representative western blots and normalized surfactant protein (SP) levels from lavage fluid and lung tissue as a function of ventilation mode as described in Figure 1. **(A)** Top: representative western blots for SP-B (~18 kDa) in the bronchoalveolar lavage (BAL), proSP-B (~40 kDa) from lung tissue, the dimer form of SP-B (~18 kDa) from lung tissue and the loading control (LC) β-actin (~42 kDa). Bottom: statistics show that SP-B in BAL increases with VV2 relative to VV1 (*p* < 0.005). The proSP-B is higher during VV2 than CV (*p* < 0.005) whereas the levels of the dimer are different for all pairwise comparisons (*p* < 0.001). **(B)** Top: representative western blots for proSP-C (~21 kDa) in BAL and lung tissue (~25 kDa) together with the β-actin LC (~42 kDa). Bottom: Compared to CV, proSP-C decreases with VV1 and VV2 both in the lavage (*p* < 0.001) and the lung (*p* = 0.006). **(C)** Top: representative western blots for SP-A (~38 kDa) in BAL and lung tissue (~26 kDa) together with the β-actin LC (~42 kDa). Bottom: Compared to CV, SP-A in the lavage decreases with VV2 (*p* < 0.001), and in the lung, it increases with VV1 and VV2 (*p* < 0.001). **(D)** Top: representative western blots for SP-D (~35 kDa) in BAL and lung tissue (~35 kDa) together with the β-actin LC (~42 kDa). Bottom: Compared to CV, SP-D decreases with VV1 and VV2 both in the lavage and the lung (*p* < 0.001) and there is also a significant difference between VV1 and VV2. ^*^ and # denote statistically significant difference from CV and VV1, respectively.

The levels of SP-B, responsible for reducing the surface tension of the air-liquid interface, are shown in Figure 3A. SP-B increased significantly in the lavage following only VV2 (*p* < 0.005) compared to VV1. The pro form of SP-B (proSP-B) in the tissue represents SP-B production by type II epithelial cells and these data demonstrate that VV2 stimulates more SP-B production compared to CV. Finally, the dimer form of SP-B was higher after both VV1 and VV2 than CV and it was also higher in VV2 compared to VV1 (*p* < 0.001). While SP-C contributes to normal surface tension at the air-liquid interface (Ingenito et al., 1999), it also has a role in inflammation (Glasser et al., 2013). In this study, proSP-C (Figure 3B) was consistently higher following CV than VV1 and VV2 both in the lavage (*p* < 0.001) and the lung (*p* = 0.006).

SP-A is an innate immune system component of SPs and it appears to promote SP-B related functions (Veldhuizen and Haagsman, 2000). SP-A decreased significantly in the lavage following VV2 compared to both CV and VV1 (Figure 3C), but SP-A was higher in the lung tissue after VV1 as well as VV2 compared to CV (*p* < 0.001). The levels of SP-D (Figure 3D), also an immune regulatory surfactant protein, were significantly higher in the lavage and the lung following CV compared to VV1 or VV2. Interestingly, VV2 secreted more SP-D into the lavage than VV1 (*p* < 0.001).

### VV2 produces the least inflammatory markers and vascular leakage

**Figure 4:**
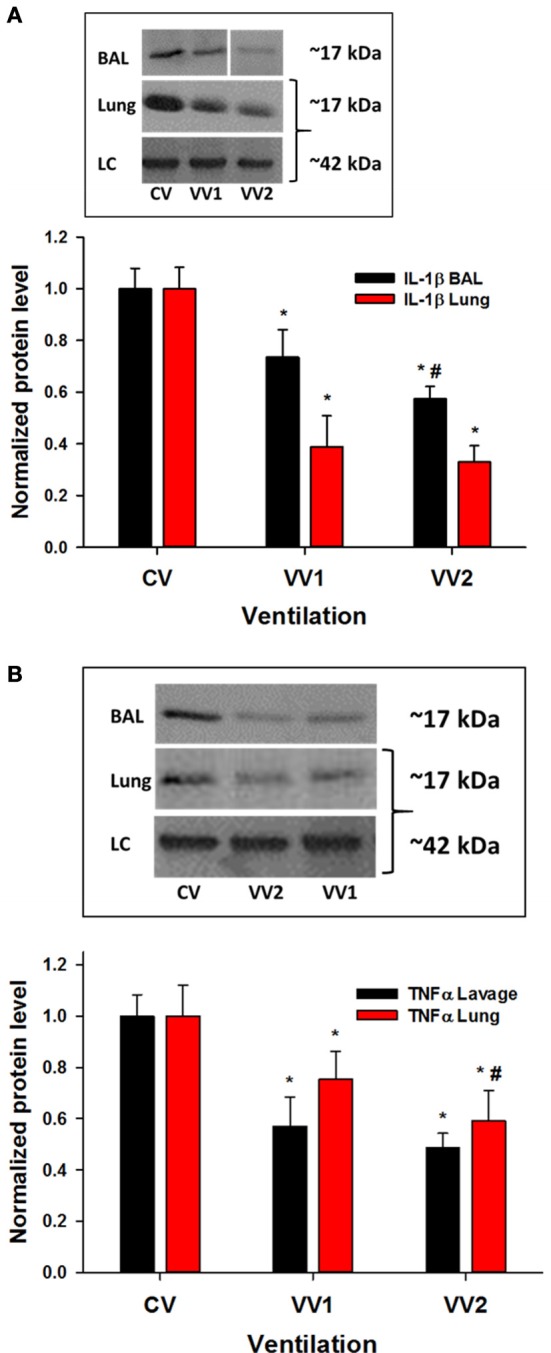
Representative western blots and the mean and SD of cytokine levels in bronchoalveolar lavage and lung tissue as a function of ventilation mode (see Figure 1). **(A)** Top: representative western blots in BAL and lung tissue for IL-1β. The IL-1β (~17 kDa) levels significantly decrease with both VV1 and VV2 compared to CV in the lavage (*p* < 0.001; all three groups are different) as well as in lung tissue (*p* < 0.001). **(B)** The TNFα (~17 kDa) (top: blots) levels are lower in the lavage following both VV1 and VV2 than CV (*p* < 0.001) while the levels from the lung tissue are different between all three groups (*p* < 0.001). LC: β-actin (~42 kDa). ^*^ and # denote statistically significant difference from CV and VV1, respectively.

The levels of IL-1β, an inflammatory cytokine, were lower following both VV1 and VV2 in the lavage (*p* < 0.001) as well as in lung tissue (*p* < 0.001) compared to CV (Figure 4A). Furthermore, IL-1β was reduced in the lavage of VV2 lambs compared to VV1. Another inflammatory cytokine, TNFα, showed reduced expression in the lavage following both VV1 and VV2 than CV (*p* < 0.001; Figure 4B). The levels of TNFα from the lung tissue were different in all three groups (*p* < 0.001) with VV2 producing the least amount.

**Figure 5:**
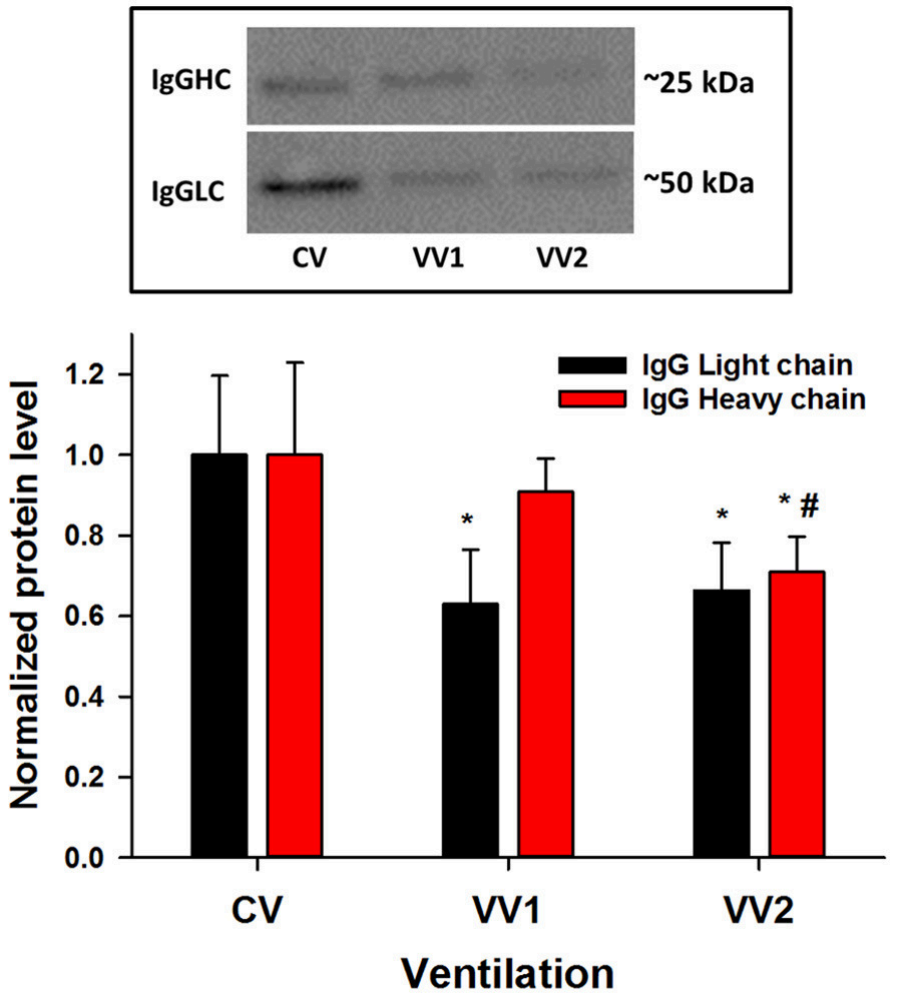
Mean and SD of the levels of immunoglobulin G light (IgGLC) and heavy (IgGHC) chains from lavage fluid as a function of ventilation mode (see Figure 1). For the light chain (~25 kDa), both VV1 and VV2 produce significantly less IgG than CV (*p* < 0.01) whereas for the heavy chain (~50 kDa), VV2 had less IgG than both CV and VV1 (*p* < 0.01). ^*^ and # denote statistically significant difference from CV and VV1, respectively.

The light and heavy chain expressions of IgG were measured in the lavage fluid to characterize whether vascular leakage occurred during ventilation (Figure 5). For the light chain, both VV1 and VV2 produced significantly less IgG than CV (*p* < 0.01). However, for the heavy chain, VV2 had less IgG than both CV and VV1 (*p* < 0.01) suggesting better protection of the endothelial-epithelial barrier.

### VV2 produced the least histological damage

**Figure 6:**
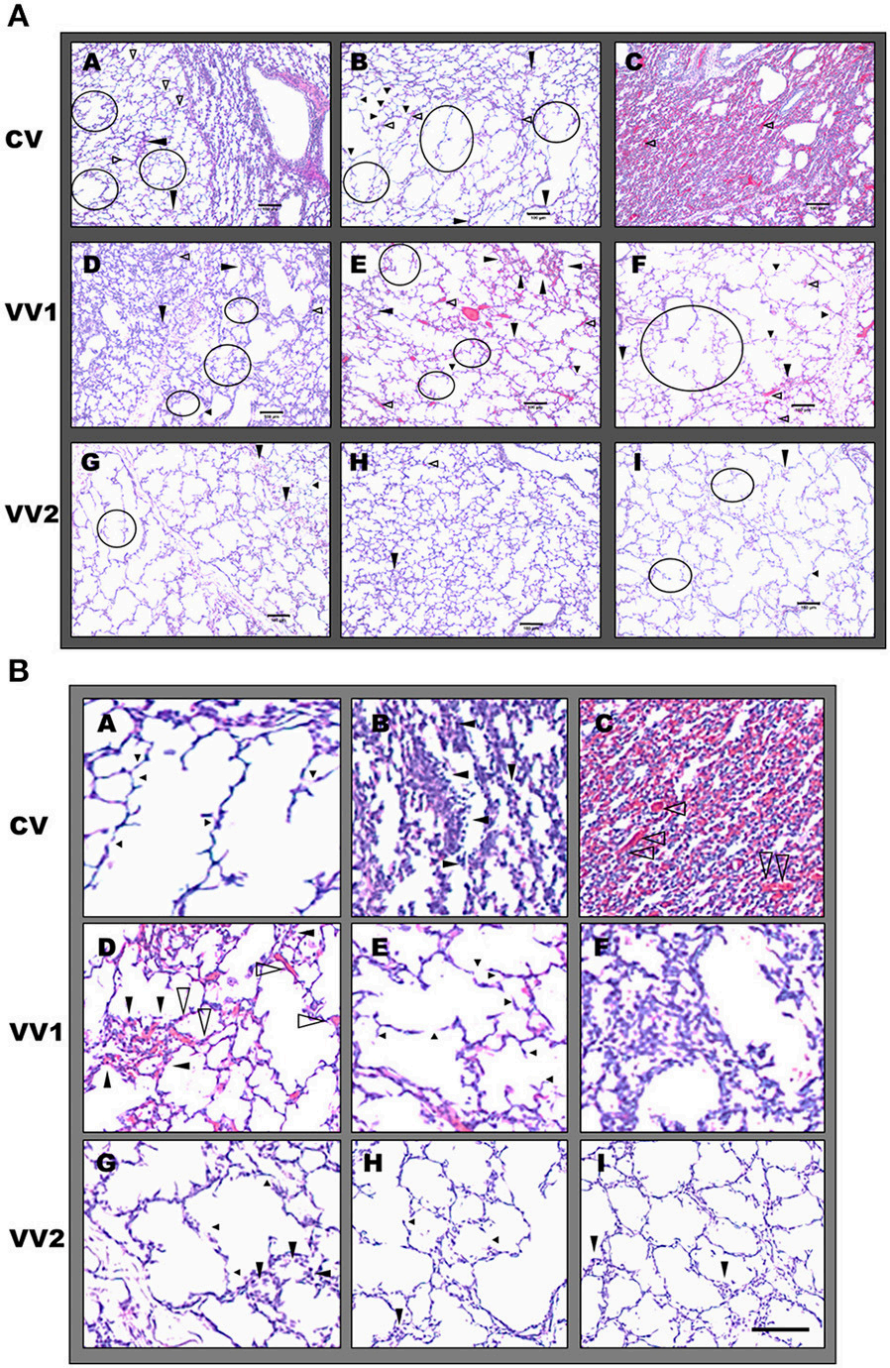
**(A)** Representative images of haematoxylin and eosin (H&E) stained pre-term lamb lung sections. A–C represent conventional ventilation (CV), D–F are from VV1 whereas G, H and I are from VV2 (see Figure 1). Large black arrows indicate blood cell infiltration, small black arrows point to weakened, damaged thinner alveolar walls and opened black arrows show dilated or congested capillaries. The walls with retracted tissue are circled in black. A, shows collapsed area near a healthy alveolar region whereas C, shows a totally collapsed region with hemorraghic vascular damage associated with CV. D represents regions associated with excessive alveolar wall thickening and inflammatory cell invasion whereas E, shows vessel damage without collapse with VV1. Alveolar weakening is present in all ventilation regimens with the lowest occurrence in VV2. Scale bar: 100 μm. **(B)** Representative H,E stained pre-term lamb lung sections with histopathological details. A–C represent CV; D–F are from VV1 whereas G–I are from VV2. A shows alveolar wall weakening and breakage; B shows excessive alveolar wall thickening and inflammatory cell invasion whereas C shows a collapsed region with hemorrhagic vascular damage associated with CV. D represents regions associated with vessel damage (dilated capillaries, inflammatory cell infiltration and small interstitial edema) without alveolar collapse, whereas E shows weakened or broken alveolar regions. In F, inflammatory cell invasion can be seen following VV1. Some alveolar weakening and inflammatory cell infiltration is also present in VV2 but with significantly reduced occurrence shown in G–I. Scale bar: 100 μm.

**Figure 7:**
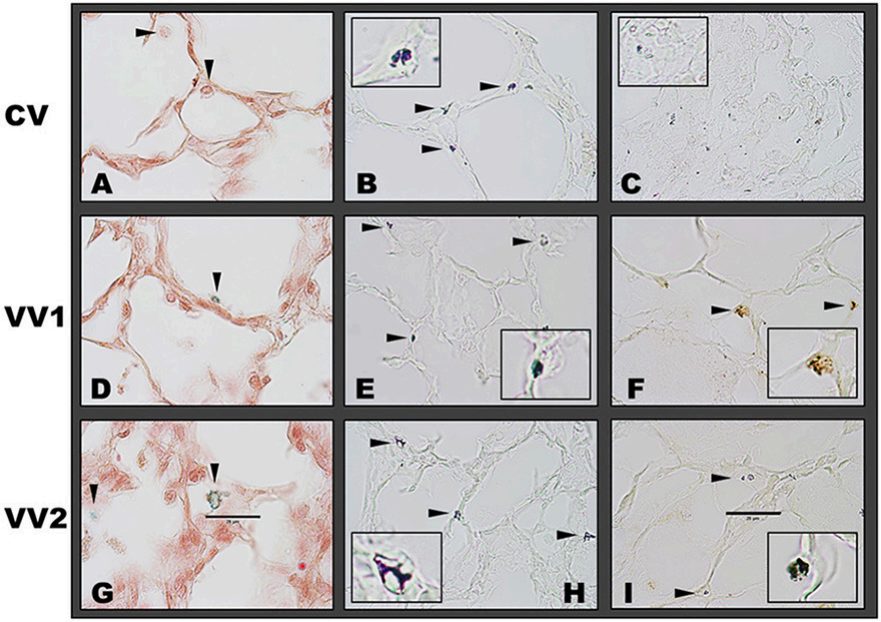
Representative images of lipid stained preterm lamb lung sections. **(A–C)** represent conventional ventilation (CV), **(D–F)** are VV1 whereas **(G–I)** are VV2 (see Figure 1). **(A,D,G)** show the phospholipid (green) staining by Pearse's method, where phospholipid positive cells are marked with black arrows. The CV group shows weak staining for phospholipids whereas VV1 and VV2 exhibit stronger staining. The neutral red pinkish counterstain shows the nuclei and lung structure. **(B,C,E,F,H,I)** show lipid (dark purple and blue) staining using Nile blue. In open lung areas, all conditions exhibit similar number of blue positive cells with dominant acidic lipids indicated by the dark blue staining with slightly larger pools in VV2. VV1 exhibits yellowish blue color indicating a larger amount of neutral lipids compared to the other two conditions, but it also contains acidic lipids indicated by the dark blue staining. However, in the collapsed area **(C)**, CV shows numerous smaller positive staining pattern. The lung tissue is unstained. Insets are magnified regions showing representative positive cells. Scale bar: 25 μm.

At the alveolar level, H&E staining showed the most intact and homogeneous structure as well as the least amount of blood cell infiltration following VV2 (Figure 6). In contrast, CV lungs exhibited alveolar over-distension with weakened and damaged thin alveolar walls and nearby collapsed and thickened alveolar regions. Vascular damage was also evident with dilated capillaries that were often surrounded by regions infiltrated with varying amounts of blood cells even reaching hemorrhagic infarction. Lambs responded to VV1 in a mixed manner with some animals showing normal alveolar structure while others showing significant heterogeneities with excessive alveolar wall thickening and infiltration of blood cells. Table 3 summarizes the histological scores. Furthermore, the images in Figure 7 demonstrate that the number and distribution of phospholipid positive cells were heterogeneous but consistently showed larger pools and more homogeneous distribution in the VV2 group. The CV group in the collapsed regions (Figure 7C) exhibited reduction in size but numerous small dots of phospholipid staining. Compared to CV and VV2, the images with the differential Nile Blue A stain from the VV1 group suggested a slightly different lipid composition with more neutral lipids present in the cells. Semi quantitative scoring of the number and size of phospholipid positive cells provided the following ranges: 2–4 for CV, 5–7 for VV1 and 7–9 for VV2 (on a scale of 0 to 10 with 0 representing no phospholipid positive cells).

**Table 3:** Injury scores based on histological evaluations.

**Condition**	**Alveolar wall thickening**	**Alveolar wall breakage**	**Blood cell infiltration**	**Interstitial edema**	**Vascular bed injury**	**Alveolar wall flooding**
CV	7–9	7–8	7	6–7	6–7	7
VV1	2–6	2–6	2–5	1–3	1–4	1–3
VV2	1–2	1–2	1	1	1	0

*Histological scoring criteria: Alveolar wall thickening: structural changes in the alveolar extracellular matrix (ECM) due to swollen and/or loose ECM and hyaline membrane formation covering the epithelial layer. Alveolar wall rupture: thinner or broken alveolar walls with tissue retraction. Blood cell infiltration: increase in inflammatory cell numbers in the alveolar wall. Interstitial edema: protein containing free fluid and swelling of the extracellular matrix of alveolar wall space. Vascular bed injury: degeneration of vessels (swollen, vacuole rich vascular walls with cell necrosis). Alveolar wall flooding: platelet invasion including infarcts like hemorrhagic changes of alveolar walls after vascular rupture. Left upper lobes of the lungs (n + 5 in each group) and 20 sections/lamb were chosen randomly for analysis*.

### Performance indexes

**Figure 8:**
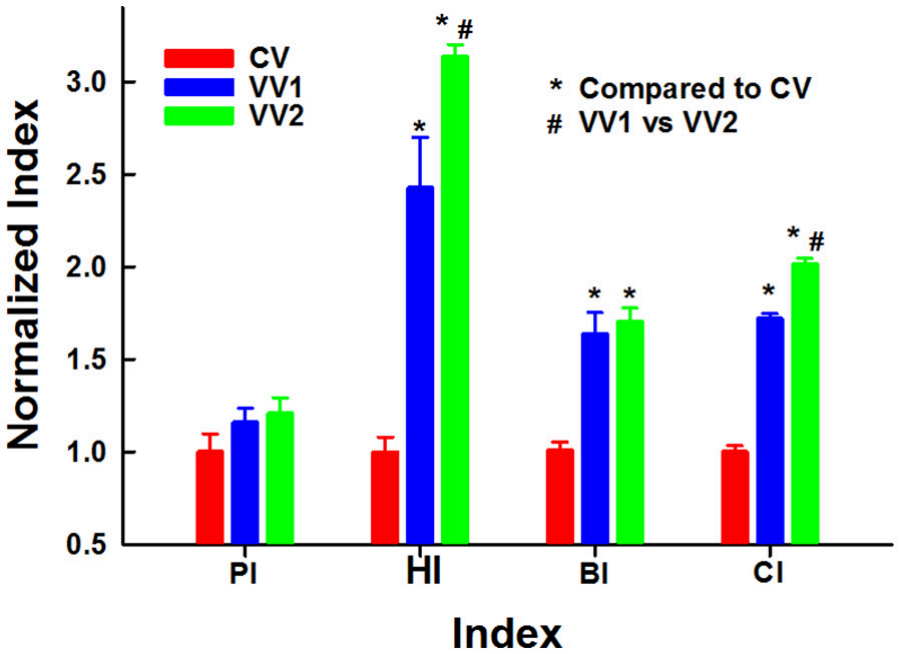
Physiology (PI), histology (HI), biochemistry (BI) and combined (CI) indexes as a function of ventilation mode (see Figure 1). ^*^ and # denote statistical significance (*p* < 0.001) compared to CV and VV1, respectively.

The mean and SD of the various performance indexes including PI, BI, HI, and CI at 180 min are summarized in Figure 8. These data demonstrate that both VVs strongly outperformed CV, except in physiology, while VV2 was clearly and statistically significantly better than VV1 in CI (*p* < 0.001).

## Discussion

This study aimed to optimize variable ventilation for best performance and to compare variable ventilation to conventional ventilation as a control in a preterm lamb model of neonatal RDS. We compared the physiological response, the surfactant proteins, two inflammatory cytokines, leakage and histology of the lung following 3 h of immediate postpartum ventilation with CV and two different implementations of VV. VV1 provides relatively small but continuous variations in *V*_T_ around the mean, whereas VV2 reaches as high as 2.25 times the mean *V*_*T*_ (Figure 1). Our main findings are as follows: (1) both VV1 and VV2 outperformed CV in several physiology parameters (Table 2) as well as in our final combined index (Figure 8); (2) only minor physiological differences were found between VV1 and VV2 (Figure 2 and Table 2); (3) there was less SP-A in the lavage, more SP-B in the lung following VV2 and more proSP-C and SP-D following CV (Figure 3); (4) there was less inflammation (Figure 4) and leakage (Figure 5) after VV2; (5) these differences resulted in better alveolar structure (Figure 6) and hence a significantly stronger overall performance (Figure 8) of VV2.

The preterm lamb model used in this study is well established and is a standard model of infant RDS (Pillow et al., 2004, 2011; Shashikant et al., 2005; Berry et al., 2012). The natural maturation of surfactant occurs around 130 days of gestation (Braems et al., 2000) and is accelerated by pre-treating ewes with betamethasone (Ballard et al., 1997). Therefore, our results provide evidence that despite the steroid treatment (1–2 days prior to delivery), ventilation mode has a major influence on lung maturation and physiology. Specifically, we found that not only the presence of breath-to-breath variability in *V*_T_, but its specific distribution regulates surfactant metabolism, inflammation, leakage and consequently lung physiology in a complex manner.

The breath-to-breath variations in *V*_T_ present in both VV1 and VV2 generate corresponding fluctuations in stretch amplitudes to which lung cells are exposed during ventilation. Since the secretion of surfactant phospholipids is accelerated by single and repeated stretches in alveolar type II cells (Wirtz and Dobbs, 1990), it may be expected that continuous variations of local stretch stimulates surfactant lipid and surface tension regulatory surfactant proteins SP-B and SP-C production and/or secretion. Indeed, our previous cell culture study showed that type II cells downregulated phospholipid secretion when cells were stretched using a monotonous stretch pattern compared to variable stretch pattern (Arold et al., 2009). Our results are consistent with the cell culture results suggesting that fluctuations in *V*_T_ stimulate lipid production (Figure 7). The mechanism by which SP-B is increased in the lavage following VV2 and the pro form and dimer of SP-B in the tissue by both VV1 and VV2 (Figure 3) is not known. A possible explanation is via extracellular ATP which is a known surfactagogue (Rooney, 2001). We reason that cycle-by-cycle variations in stretch upregulates intracellular ATP production by doubling its level through a microtubule- and non-muscle myosin-dependent pathway compared to monotonous stretch, in a similar manner to our observation in vascular smooth muscle cells both in culture and intact tissue (Bartolak-Suki et al., 2015). Consequently, extracellular ATP concentration may increase following VV. Indeed, type I alveolar cells increase the release of extracellular ATP threefold in response to stretch that increases surfactant secretion by type II cells (Patel et al., 2005). An alternative or perhaps complementary explanation is that variable stretch during VV2 lowers SP-A secretion (Figure 3C), and since extracellular SP-A inhibits lamellar body secretion (Bates et al., 2003), SP-B secretion is less inhibited by VV2. The elevated level of proSP-B following VV2 is consistent with enhanced production compared to both CV and VV1 which may be related to stronger signaling to the nucleus via cytoskeletal reorganization following VV (Bartolak-Suki et al., 2015). Alternatively, the increased level of proSP-C following CV (Figure 3B) may be more related to the immunomodulatory role of SP-C (Mulugeta and Beers, 2006) than its contribution to surface tension. Indeed, SP-D was also higher in the CV group than in the VV groups (Figure 3D). Another possible explanation for the increased SP-C in CV could be related to balancing the lipid components of surfactant by excluding non-dipalmitoylphosphatidylcholine lipids from the alveolar interface during expiration strengthening the attachment of the bilayer structure to the lipid monolayer (Veldhuizen and Haagsman, 2000). This in turn may compensate for the reduction in SP-B. The mechanism of lipid production, maturation and secretion during VV is not well known and warrants further studies.

The improvement in SP-B metabolism by VV2 compared to VV1 may be related to the presence of intermittent larger stretch amplitudes in VV2 (Figure 1) which can be considered as occasional but randomly occurring small recruitment maneuvers. Although a larger variability around the mean stretch amplitude stimulates phospholipid secretion (Arold et al., 2009), VV2 is not equivalent to simply adding periodic recruitment maneuvers to CV (Thammanomai et al., 2008). Instead, we believe it is the nonlinearities of the mechanotransduction pathway combined with the stochastic nature of the stretch stimulus (Suki et al., 2016) that leads to the differential regulation of SPs by VV1 and VV2. CV without variability also allows SP-B secretion that is only 8% lower than during VV (Figure 3) although the difference is statistically significant. However, CV does not stimulate the maturation of the dimer form and induces the lowest level of proSP-B in the lung and hence CV does not replenish SP-B for the secretion. Thus, our results demonstrate that stretch pattern does govern the production, storage and secretion of SP-B and that these processes were more optimal during VV2 with sustained secretion but without depletion of SP-B stores due to production by type II cells (Figure 3A). Stretch pattern also influenced the secretion of SP-A with the lowest level during VV2 (Figure 3C) and proSP-C (Figure 3B) as well as SP-D (Figure 3D) with highest levels following CV. These findings likely represent reduced inflammation that is also supported by the low levels of TNFα and IL-1β (Figure 4) following VVs. Additionally, in the lung, the elevated level of SP-A during VV compared to CV suggests that stretch pattern helps mature the innate immune system since TNFα and IL-1β levels were consistently low.

The different amounts of surfactant proteins in the tissue and at the air-liquid interface following the three ventilation modes have important consequences for inflammation, leakage and lung function. First, application of CV induced an upregulation of both IL-1β and TNFα mRNA in lung tissue in similar gestation preterm lambs (Naik et al., 2001) as in the present study. We also found higher protein levels of both IL-1β and TNFα in the BAL and lungs in CV compared to both VVs (Figure 4). Additionally, IL-1β increased the expressions of both SP-A and SP-B in premature rabbit lungs (Vayrynen et al., 2002). In contrast, SP-A increased neutrophil infiltrates and accumulation of IL-1β but not TNFα in the lung after 6 h of ventilation in preterm lambs, whereas adding SP-A to surfactant treatment of preterm lambs had no effects on lung function (Kramer et al., 2001). In our study, histological analysis showed more inflammatory cell infiltration in the CV and VV1 groups than in the VV2 group (Figure 6). Since lung collapse amplifies cytokine expressions in the preterm lung (Naik et al., 2001), the fact that VV2 had the least amount of collapse (Figure 6) likely contributed to the lower inflammation compared to both CV and VV1.

The lower levels of IgG light chain during both VVs compared to CV suggest that VV provides better protection than CV (Figure 5) despite the compromise of the epithelial-endothelial barrier (IgG is not expected to be found in the lavage). However, the presence of significantly more IgG heavy chain following VV1 than VV2 is interesting from the point of view of optimizing VV. We argue that VV2 was able to maintain a more open lung (Figure 6) partly due to more SP-B (Figure 3A) and hence a reduced surface tension of the air-liquid interface and partly due to direct nonlinear effect of variations in V_T_ that is more effective in recruitment than CV (Suki et al., 1998). An alternative explanation is that VV1 promotes more regional collapse and alveolar instability which in turn leads to repetitive opening and closing of alveoli (Muscedere et al., 1994) with high shear and normal stress gradients (Bilek et al., 2003) that may compromise the epithelial-endothelial barrier. It is important to notice that animals in the VV1 group had the most heterogeneous response in terms of both biochemistry and histology with some animals having normal surfactant and tissue structure while others showing significant damage. This heterogeneity suggests that the individual pre-ventilation maturation history of the animal can greatly influence the outcome of ventilation during VV1.

The above biochemical and structural changes in the lung explain the superior physiological results following either VV ventilation pattern compared to CV (Table 2). It is interesting to note that even though *Pa*CO_2_ was a target variable, it was lower during VV2 compared to CV (Table 2). This finding indicates that it was more difficult to keep *Pa*CO_2_ within a target range during CV despite the comparable *V*_T_s in VV2. The difference in the performance between VV1 and VV2 was not striking in terms of the individual physiological variables (Table 2). While VV1 had slightly lower overall *V*_T_ than CV, VV2 had lower *P*aCO_2_ and C_*s*_ suggesting a better improvement in physiology over CV. This slight improvement in physiology together with the substantially better surfactant metabolism, barrier protection and lung structure manifested in a much better overall combined index for VV2 (Figure 8). Over a longer ventilation protocol this improved efficacy could further differentiate between VV1 and VV2. Finally, the improved cerebral tissue oxygenation seen in both VV ventilation groups (Table 2) may have important implications for future ventilation strategies for premature infants to protect against both bronchopulmonary dysplasia and adverse neurodevelopmental outcomes.

In summary, ventilating preterm lambs with VV has the potential to be a therapeutic tool and hence it has significant benefits compared to CV. Our results also suggest that VV can be further optimized to enhance its performance that may help weaning critically ill patients from mechanical ventilation. Nevertheless, before VV can be used in human infants, these results need to be extended to longer term ventilation periods and compared to other lung protective ventilation modes such as high frequency oscillatory ventilation.

## Author contributions

EB, BS, and JP contributed to the design of the studies. EB and BS prepared and drafted the manuscript. EB, BS, PN, and JP carried out the physiological, biochemical, and histological studies. EB, BS, SB, and JP interpreted the experimental data. All authors performed critical revision of the manuscript for intellectual content.

### Conflict of interest statement

The authors declare that the research was conducted in the absence of any commercial or financial relationships that could be construed as a potential conflict of interest.
